# Cardiovascular Exercise Drives Neuroprotection in a Mouse Model of Spinocerebellar Ataxia 1 Via Rescue of Aberrant Splicing

**DOI:** 10.1002/ana.78282

**Published:** 2026-06-17

**Authors:** Isabel Soto, Michael Bonomo, Brianna S. Nelthrope, Benjamin D. Carr, Emma Palmer, Mariana Sierra, Ilkim Erturk, David Peppercorn, Eleonor Cox, Tiare K. Vasconcellos, Henry L. Paulson, Hannah K. Shorrock, Sharan R. Srinivasan

**Affiliations:** ^1^ Department of Neurology University of Michigan Ann Arbor MI USA; ^2^ Department of Biological Sciences and the RNA Institute, College of Arts & Sciences University at Albany, State University of New York Albany NY USA; ^3^ Program in Biomedical Sciences University of Michigan Ann Arbor MI USA

## Abstract

**Objective:**

Spinocerebellar ataxia 1 (SCA1) is a fatal hereditary neurodegenerative disorder with no approved therapies, and gene‐targeting strategies have thus far failed in clinical trials. Exercise remains the only intervention shown to provide clinical benefit in patients with spinocerebellar ataxias (SCAs), yet the underlying mechanisms remain poorly understood.

**Methods:**

Using the Atxn1^154Q/2Q^ knock‐in mouse model, we implemented a prolonged voluntary wheel‐running paradigm from 4 to 16 weeks of age. Mice were then phenotyped using motor and cognitive behavioral assays. Following euthanasia, cerebellar tissue was harvested for histological analysis and unbiased transcriptomics.

**Results:**

Unrestricted exercise rescued motor ataxia but not degeneration in this SCA1 mouse model at the ages in this study. Transcriptional profiling of cerebellar tissue separated wild‐type (WT) sedentary and exercise mice by differential gene expression, but in SCA1 mice the most pronounced changes occurred at the level of RNA splicing, particularly in ion channel modules. Exercise led to a significant rescue of splicing events in SCA1 mice, with minimal impact on gene expression changes. Further, both exercised SCA1 and WT mice exhibited splicing patterns more similar to each other than their sedentary counterparts.

**Interpretation:**

Together with emerging evidence in other SCAs, our findings confirm aberrant splicing as a central driver of SCA pathophysiology and identify splicing‐regulated networks as actionable therapeutic targets. The observed benefits in SCA1 mice suggest that correcting mis‐spliced ion channels may be a viable strategy for disease modification across SCAs and related neurodegenerative disorders. ANN NEUROL 2026;100:850–866

The spinocerebellar ataxias (SCAs) are a group of fatal hereditary neurodegenerative disorders characterized by gait imbalance, motor incoordination, slurred speech, and early mortality.^1^, ^2^, ^3^ In addition to these motor symptoms, a significant subset of patients experiences varying degrees of cognitive impairment.^4^, ^5^, ^6^, ^7^ Despite the severity of these conditions, no approved therapies exist, underscoring the urgent need for effective interventions. As SCAs are monogenic disorders, significant efforts have focused on targeting pathogenic gene products using approaches such as antisense oligonucleotides (ASOs).^8^, ^9^, ^10^, ^11^ However, challenges such as patient selection, timing, and intrathecal administration may limit widespread application. This highlights the necessity for alternative approaches that will be effective across a broader patient population at varying stages of disease.

To date, sustained cardiovascular exercise is the sole intervention shown to improve motor performance in patients with SCA both in acute intervention and over 1 year.^12^, ^13^, ^14^, ^15^, ^16^, ^17^, ^18^, ^19^, ^20^ In particular, aerobic exercise was shown to be superior to balance training in improving Scale for Assessment and Rating of Ataxia (SARA) scores in patients with SCA.^12^ As physical exertion is often challenging and ultimately unfeasible for patients with ataxia, pharmacological strategies replicating the neuroprotective effects of exercise would be a valuable therapeutic alternative. This opportunity prompts use of ataxia model systems where detailed analyses of the molecular effects of exercise can be pursued. Thus far, studies in SCA animal models, although relatively few, have yielded promising results. For example, in spinocerebellar ataxia 1 (SCA1) mouse models, 2 different studies using a 4‐week exercise regimen found an increase in cerebellar epidermal growth factor (EGF), increased Purkinje cell (PC) rate of survival, and an extended lifespan, but notably mixed results on motor recovery.^21^, ^22^ In SCA6 mice, exercise enhanced motor coordination and preserved PC firing while increasing cerebellar levels of brain‐derived neurotrophic factor (BDNF).^23^ Although these findings are compelling, a comprehensive and unbiased investigation of the pathways mediating exercise‐induced neuroprotection remains lacking.

In this study, we used the well‐validated SCA1 (Atxn1^154Q/2Q^) knock‐in mouse model to examine the impact of a prolonged, low‐intensity voluntary exercise regimen.^24^, ^25^ This extended 12‐week protocol allowed us to assess whether sustained exercise could produce more robust functional benefits than those observed in earlier studies that used shorter or milder interventions. To explore the underlying mechanisms, we performed unbiased transcriptomic analyses to define how exercise influences molecular pathways in SCA1. We found that exercise improved motor and cognitive performance in SCA1 mice and significantly modulated gene‐splicing profiles, highlighting the therapeutic potential of physical activity and providing new insight into splicing‐related molecular mechanisms underlying its benefits.

## Methods

### Animals

Atxn1^154Q/2Q^ (SCA1) and wild‐type (WT) C57BL/6J littermate male and female mice were obtained from Jackson Laboratory (strains 5,601 and 664, respectively). The mice were maintained on a 12‐hour light/dark cycle. All mice had ad libitum access to food and water. Behavioral tests were conducted during the light phase of the cycle, beginning when the mice were 16 to 17 weeks old (hereafter referred to as 16 weeks). All experimental procedures were approved by the Institutional Animal Care and Use Committee (IACUC) at the University of Michigan. All behavioral testing was conducted within 1 week.

### Genotyping

Genomic DNA was extracted from mouse tail samples (Qiagen DNeasy Blood and Tissue Kit). The following primers were used to confirm the presence of the expanded *Atxn1* allele: Forward (GTGAGTTTGGGTCTGGCATC); and Reverse (CCAAAAGTTAGGATCACAGCC C). To measure the expanded *Atxn1* repeat length, the following primers were used: Forward (5′ CGTGTACCCTCCTCCTCAGT 3′) and Reverse (5′ATTGCACAACCACCTGGGAT 3′). CAG repeat sizing was confirmed using fragment analysis. Each sample was diluted with Hi‐Di Formamide (Applied Biosystems), and 1,200 LIZ ladder and plated on 96‐well Micro‐amp plate (Applied Biosystems). Capillary electrophoresis was then performed on a SeqStudio Genetic Analyzer (Applied Biosystems) using the long‐fragment run module. The resulting electropherogram was used to calculate the repeat expansion by taking the larger (expanded allele) peak size and subtracting the smaller (WT allele) peak size, dividing by 3, and adding 2 to account for the WT allele.

### Exercise Protocol

Mice in the exercise groups were solo housed and given unrestricted access to vertical running wheels (KAYTEE Comfort Wheel, 14 cm diameter) for 12 consecutive weeks starting at 4 and ending at 16 weeks of age. An externally attached magnet allowed tracking distance traveled and running speed using commercial odometers (CATEYE Velo 9). Running wheels were removed from the cage 72 hours prior to behavioral testing to avoid fatigue. Mice in the sedentary group were group‐housed after finding no difference versus solo‐housed mice with access to a fixed wheel (Supplementary Fig S1).

### Motor Functioning

Behavioral phenotyping was performed within 1 week. All mice were tested in order from least to most intensive activity to avoid fatigue, proceeding from open field testing, Barnes Maze (see below), balance beam, and rotarod. Locomotor activity and anxiety‐like behavior were assessed using open‐field testing (Photobeam Activity System, San Diego Instruments), where mice explored an empty chamber for 30 minutes while beam breaks were recorded and analyzed by zone (center vs periphery). For the balance beam, mice were trained and tested over 3 days to traverse a 45 cm, 1.9 cm diameter beam to a dark goal box; latency to cross was averaged per day over 2 trials. A fall was recorded as 30 seconds, which was also used as maximum latency. On the rotarod (Med Associates ENV‐574M), mice underwent 3 daily trials for 4 days, with the rod accelerating from 4 to 40 rpm over 300 seconds. Latency to fall was recorded, and trials were stopped if mice clung without walking for 3 full revolutions. A 5‐minute inter‐trial interval minimized fatigue.

### Cognitive Functioning

The Barnes Maze was used to assess spatial learning and memory in mice.^26^ Mice were tested on a 1 m diameter, elevated maze with 20 evenly spaced 5 cm holes; one hole led to a removable escape chamber. Testing included habituation (1 day, 3 minutes of free exploration), training (3 days, 4 trials/day, 3 minutes/trial, 30 minutes inter‐trial interval), and a probe trial (72 hours post‐training). Mice failing to locate the escape chamber within the allotted time were gently guided to it and allowed an approximately 15 seconds inside. During the probe test, the escape chamber was removed, and the mice were given 60 seconds to locate the previously designated escape hole. Both training and probe trials were video recorded for mouse tracking (ANY‐Maze). The Barnes Maze Unbiased Strategy (BUNS) classification tool was used to determine search strategies used on training days.^27^

### Tissue Collection

All mice were anesthetized and transcardially perfused with sterile phosphate‐buffered saline (PBS) following final behavioral testing. The brain was bisected, with half of the tissue immediately frozen in liquid nitrogen and stored at −80°F until further processing. The remaining hemisphere was fixed in 4% paraformaldehyde (PFA) for 24 to 48 hours and then submerged in a 30% sucrose solution for 24 to 48 hours. Tissue was then embedded and frozen in optimal cutting temperature (OCT) compound (Fisher Healthcare) in the sagittal plane.

### Immunofluorescence

Then, 20 μm sections were taken from frozen embedded tissue (Leica CM1850 cryostat) and washed in methanol and PBS before permeabilizing with PBS and Triton X‐100. Slides were blocked with Mouse‐on‐Mouse Blocking Reagent (Vector Laboratories) and then stained for calbindin (rabbit, Proteintech 14470‐1‐AP) and mounted with VectaShield (Vector Laboratories). Confocal 20x Z‐stack images of the anterior (between lobules 3 and 4) and posterior (between lobules 8 and 9) of the cerebellum were taken using a Leica STELLARIS Confocal Microscope. Molecular layer thickness (MLT) and calbindin intensity was quantified in ImageJ software as previously published.^28^ Intensity measurements were normalized to average intensity per batch stained. All confocal settings were kept identical throughout imaging, which was performed within 8 weeks to minimize effects of laser lifespan.

### RNA Extraction, RNA Sequencing Data Analysis, and Data Availability

Total RNA was extracted from the cerebellum using TRIzol reagent (Invitrogen). The tissues were first homogenized with RNase‐free beads and then processed using the RNeasy kit (Qiagen), followed by DNase treatment.

All RNA samples were first assessed for quality using the DNF‐471 RNA Kit on the 5,300‐fragment analyzer (Agilent), yielding an RNA integrity number (RIN) score above 8 for all samples except 2. For these 2 exceptions (marked with * in Supplementary Table S1), fragmentation time was adjusted to 7 minutes, and polymerase chain reaction (PCR) amplification was adjusted to 11 cycles for the RNA sequencing (RNA‐seq) library preparation. RNA‐seq libraries were prepared using the NEBNext Ultra II Directional RNA Library Prep Kit for Illumina with NEBNext rRNA Depletion Kit (New England Biolabs). A total of 250 ng of input RNA was used per sample, with a fragmentation time of 15 minutes. Manufacturer protocols were followed with the following exceptions: 40 × adaptor dilutions were used, and library amplification was carried out for 13 cycles. NEBNext Sample Purification beads were used for sample clean up. Libraries were then pooled in equimolar amounts and quantified using the NEBNext Library Quant Kit for Illumina (New England Biolabs). Quality of these libraries were checked via capillary electrophoresis on the 5,300‐fragment analyzer using the DNF‐474 HS NGS kit (Agilent). Libraries were then loaded onto P2 flow cell (Illumina) and sequenced using 151 bp paired end reads on an Illumina NextSeq 2000 sequencer at the University of Albany, RNA Institute Advanced Transcriptomic Facility. All datasets have a total read depth greater than 90 million reads (see Supplementary Table S1).

FASTQ file quality was assessed using FastQC (version 0.12.1). FASTQ files with >20% adapter content was trimmed using fastp (version 0.24.0) and quality re‐confirmed using FastQC. Trimmed FASTQ files were aligned to the GRCm39/mm39 mouse reference genome using STAR (version 2.7.10a). In RStudio (2025.02.28; R software version 4.4.3), differential gene expression analysis was performed using DESeq2 (version 1.46.0) and genes that passed a threshold of adjusted *p* values (*p*‐adj) < |0.5| and a log2fc > |0.5| were considered significantly differentially expressed. For differentially expressed genes (DEGs), no events met a “responsive to exercise” parameter of a log2FC > |0.5| and *p*‐adj < 0.05 between WT sedentary and SCA exercise with a log2FC < |0.25| between WT sedentary and SCA sedentary.

Alternative splicing analysis was performed using rMATS (version 4.1.2) and events were considered significantly mis‐spliced if the false‐discovery rate (FDR) < 0.1 and ΔPSI (change in percent spliced in) > |0.1|. All ΔPSI values are converted from a ratio to a percentage with the threshold adjusted accordingly: ΔPSI > |10%|. Exon numbers are referred to by previously published exon numbers for the same coordinates or based on counting the first exon in a gene as exon 1. For splicing, events were considered rescued if ΔPSI > |10%| and FDR < 0.1 between WT sedentary and SCA sedentary, and ΔPSI > |10%| and an absolute PSI > |5%| between SCA sedentary and SCA exercise. Rescued events were considered significantly rescued if the FDR < 0.1 for SCA sedentary compared to SCA exercise. Events designated as “responsive to exercise” are defined as those with a ΔPSI < 0.1 and a *p* value > 0.05 between WT sedentary and SCA sedentary and an FDR < 0.1 and ΔPSI ≥ |0.1| for SCA exercise compared to WT sedentary.

Principal component analysis (PCA) was performed in R software with the prcomp function from the stats library (version 4.5.2) with scale = TRUE and center = TRUE, then plotted with ggplot2 (version 4.0.2). In RStudio, quadrant plots were generated using the package scales (version 1.3.0). Gene ontology was assessed through Metascape GO (version 3.5.20250101).

#### Cell Type Specific Expression Analysis

Genes in which skipped exon events were dysregulated between sedentary WT and SCA1 mice (888 skipped exon [SE] events) as well as genes where splicing dysregulation was rescued by exercise (598 events) were used as 2 independent input lists for cell type specific expression analysis. Before cross comparison with single cell RNA sequencing data, event lists were converted from individual SE events to genes impacted by differentially regulated SE events such that genes in which multiple SE events were dysregulated only appeared once; this resulted in 725 genes for sedentary WT versus SCA1 and 498 genes with rescued SE events. The adult cerebellum single cell dataset was downloaded from the Single Cell Portal hosted by The Broad Institute, then manually subsetted to include only the genes from one of the 2 above lists.^29^, ^30^ Not all genes impacted by SE events were present in the single cell RNA sequencing dataset, as such, the final data for this analysis is compiled from 660 genes for sedentary WT versus SCA1 and 457 genes with rescued SE events. For each cell, the average expression of genes from the input list were averaged, and then cells were grouped by cluster annotation as input for violin plots. This method is consistent with that used to generate violin plots for gene expression in the single cell portal but allows a larger number of genes to be investigated simultaneously.^30^

#### Quantitative PCR and Splicing Analysis

Quantitative PCR (qPCR) was performed using multiplexed TaqMan probes for *Calbindin* (FAM; Thermo Fisher, Mm00486647_m1) or *Kcnma1* (FAM; Thermo Fisher, Mm01268569_m1), normalized to *Actb* (VIC; Thermo Fisher, Mm02619580_g1). All reactions were run in technical triplicate on a QuantStudio 6 Flex Real‐Time PCR System (Applied Biosystems).

For confirmatory splicing analysis, 250 ng total RNA from each sample was reverse transcribed using SuperScript IV reverse transcriptase (Invitrogen) and random hexamers (IDT). Selected splicing events were assessed by PCR amplification. Primer sequences, annealing temperatures, and product sizes are as follows: Cacna2d2 exon 23 Forward (5′ AGCCAACCTCAGCGACCA 3′), Reverse (5′ TGAAAACGTGTCCTTCAGACTC 3′), Ta 54.9°C, inclusion 107 bp, exclusion 86 bp. Trpc3 exon 9: Forward (5′‐CTAACTTTTCCAAATGCAGGAGGAGAAG‐3′); Reverse (5′ TCGCATGATAAAGGTAGGGAACACTAGA‐3′), Ta 57.7°C, inclusion 501 bp, exclusion 417 bp. Kcnma1 exon 23 Forward (5′ GGACAGATCATCACCCGACA 3′), Reverse (5′ ACAAGCAAAGGGCTGTGTGA 3′), Ta 56.6°C, inclusion 260 bp, exclusion 180 bp.

PCR products were resolved by capillary electrophoresis performed on a SeqStudio Genetic Analyzer (Applied Biosystems). Each sample was run in triplicates and diluted with Hi‐Di Formamide (Applied Biosystems) and LIZ ladder (Thermo‐Fisher) on a 96‐well Micro‐amp plate. The resulting electropherogram was then used to calculate the ratio of the spliced in product compared to total product using the Thermo‐Fisher Connect online software.

### Statistical Analysis

Statistical analysis was performed using GraphPad Prism (version 10). A repeated measures 2‐way ANOVA was used to determine genotype and exercise effects on odometer, rotarod, balance beam, and Barnes maze results. For Barnes Maze testing, intra‐group comparisons between day 1 and day 3 were performed to assess learning capacity and inter‐group differences assessed on day 3 only. A 2‐way ANOVA was used to determine genotype and exercise effects in open field testing (OFT), probe‐trial of Barnes Maze, MLT measurements, calbindin intensity, calbindin and *Kcnma*1 gene expression, and splicing. Bonferroni‐corrected post hoc comparisons were performed between WT sedentary and SCA1 sedentary, WT sedentary and WT exercise, SCA1 sedentary and SCA1 exercise, and WT exercise and SCA1 exercise. A Shapiro–Wilk test was used to assess data normality, followed by a Grubb's test to identify any outliers (alpha = 0.05), based on the number being evaluated. All *p* values < 0.05 were noted as statistically significant.

To assess the validity of observed clustering in the PCA plots, cluster analysis was performed with the PERMANOVA package in R software (version 0.2.0), with the PostHocComp function used for post hoc comparisons between all groups. Prior to PERMANOVA analysis, homogeneity of multivariate dispersions was tested with PERMDISP, implemented in the betadisper function from the vegan package (version 2.7.2). It is important to note that whereas PERMDISP identifies dispersion effects within the data; if PERMDISP is significant, PERMANOVA cannot distinguish between only dispersion effects being present or differences in both dispersions and centroid locations.^31^ Euclidean distances between each centroid were calculated with the dist_between_centroids function from usedist (version 0.4.0), and the distance for each individual sample to its centroid were calculated and averaged in R software. Euclidean distances for alternative splicing analysis represent PSI values (minimum of 0 and maximum of 100), and for differential gene expression represent DESeq2 (version 1.50.2) counts normalized using the vst() function (minimum of 5.167 and maximum of 18.819).

## Results

### SCA1 and WT Mice Have a Similar Exercise Capacity

**FIGURE 1:**
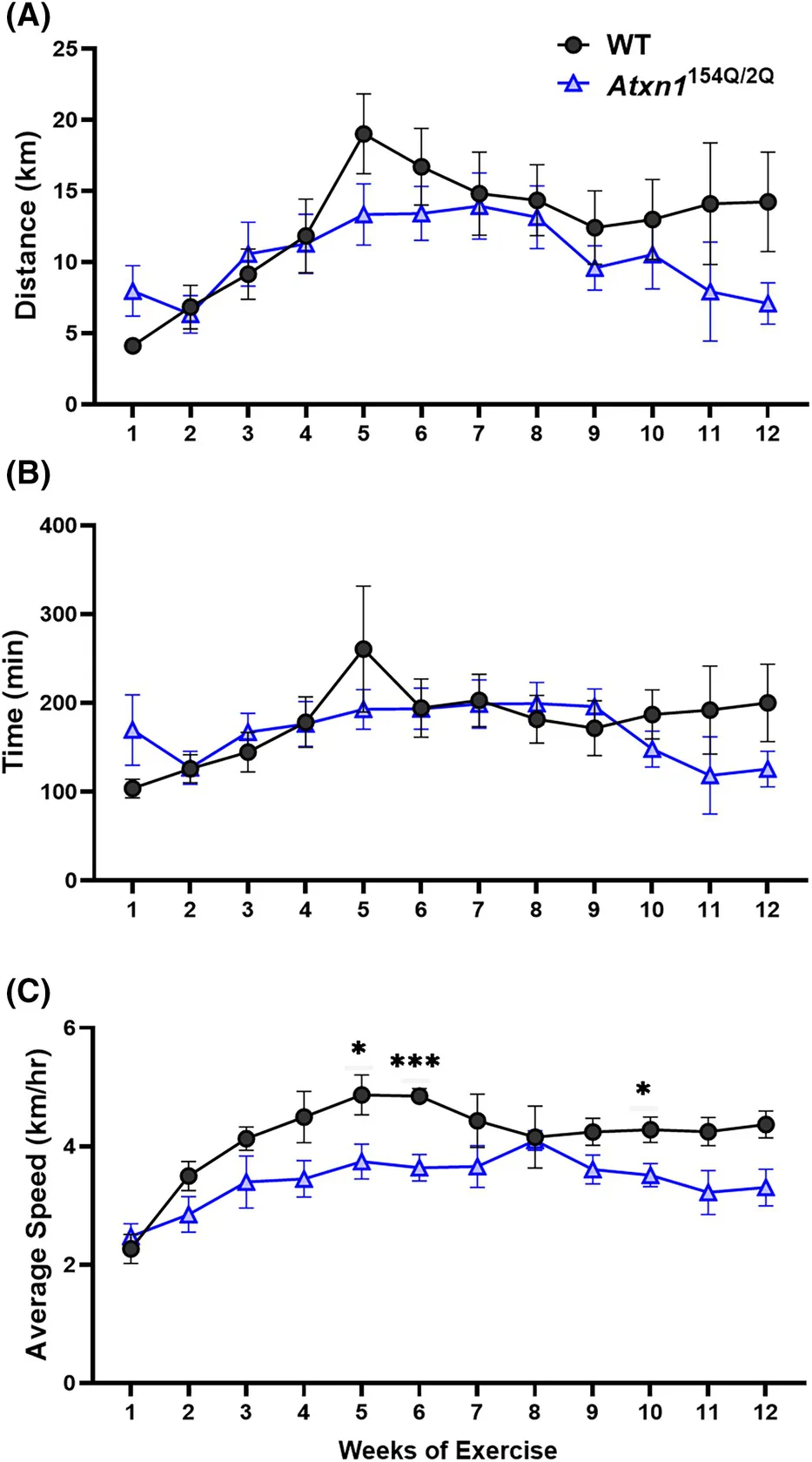
Wheel‐running activity in WT and *Atxn1* ^154Q/2Q^ mice. (A) Average running time using in‐cage running wheels did not differ between genotypes (*F*(1, 20) = 0.004, ns), and (B) *Atxn1* ^154Q/2Q^ mice reached similar peak speeds as WT mice (*F*(1, 20) = 3.973, ns). (C) *Atxn1* ^154Q/2Q^ mice consistently ran slower than WT mice (*F*(1, 20) = 8.226, *p* = 0.0095). Data represent mean ± SEM. N = 10 WT and N = 12 SCA1 mice. Weeks of exercise indicate time after initiation at 4 weeks of age. ns = not significant; WT = wild‐type. [Color figure can be viewed at www.annalsofneurology.org]

Using cage‐mounted odometers, we tracked the duration, average speed, and maximum speeds for each individual mouse in the exercise arm of our study, from 4 to 16 weeks of age. As indicated in Figure 1, we found no significant difference between SCA1 and WT mice in the average duration of exercise (A) or maximum speed (B). There was, however, a significant genotype main effect in average speed (C, *p =* 0.0095) with SCA1 mice exercising at a lower average speed than WT mice.

### Unlimited Voluntary Cage Running Rescues Motor Ataxia in SCA1 Mice

**FIGURE 2:**
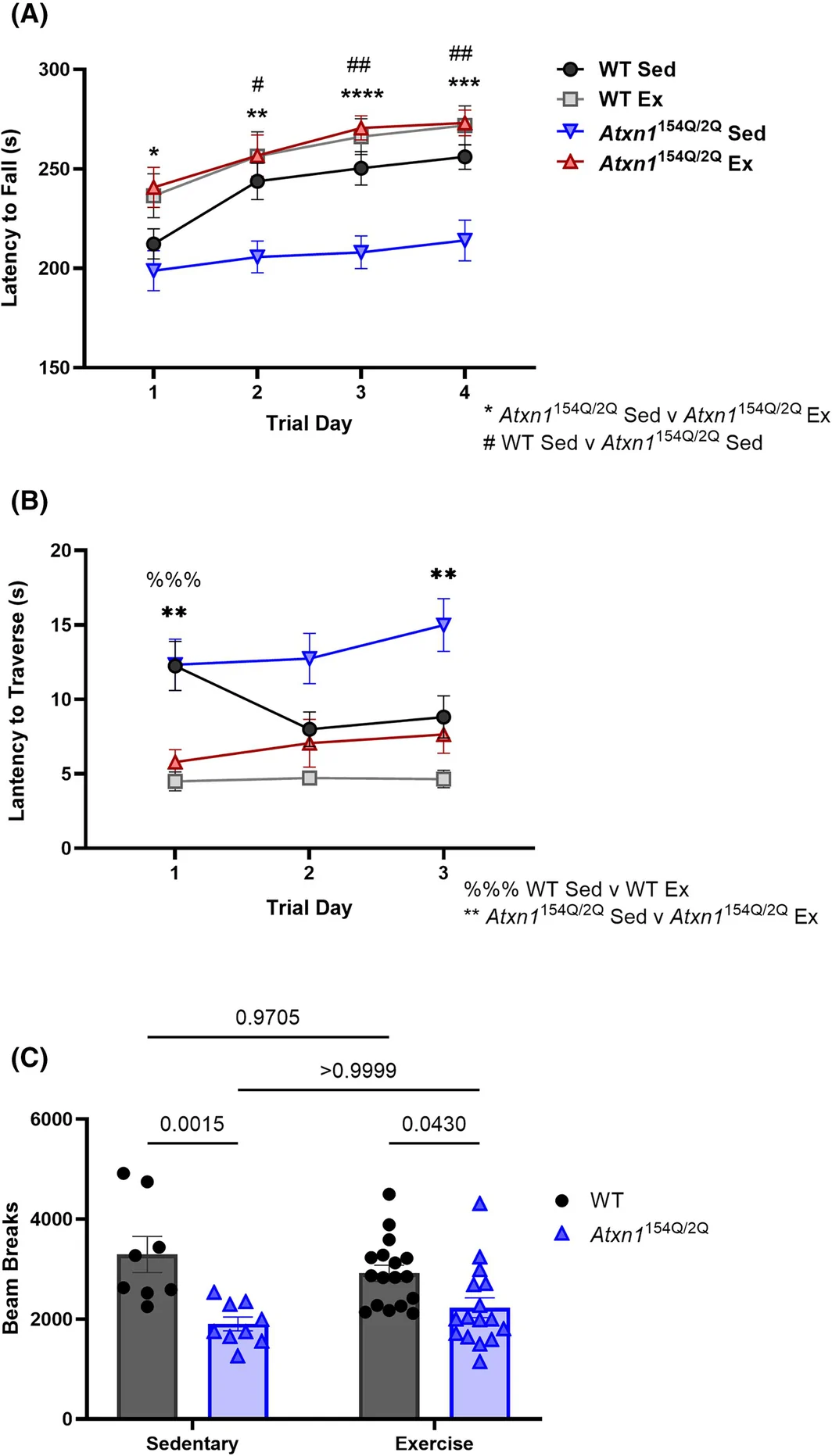
Exercise improves motor coordination and balance in *Atxn1* ^154Q/2Q^ mice. (A) Exercised *Atxn1* ^154Q/2Q^ mice performed superior on the rotarod compared with sedentary *Atxn1* ^154Q/2Q^ mice (trial day: (F(2.6, 152.5) = 19.55, *p* < 0.0001); group effect: (*F*(3, 59) = 12.24, *p* < 0.0001); training day × group (*F*(9, 177) = 1.07, ns; *p < 0.05, **p < 0.01, ***p < 0.001, ****p < 0.0001; #*p* < 0.05, ##*p* < 0.01). Additional significant differences: WT sedentary day 1 versus 3, *p =* 0.01, day 1 versus 4 *p =* 0.003; WT exercise day 1 versus 3 *p =* 0.048. (B) Exercised *Atxn1* ^154Q/2Q^ also crossed the elevated balance beam significantly faster than sedentary *Atxn1* ^154Q/2Q^ mice (training day: (F(1.92, 163.2) = 0.54, ns); group effect: (F(3, 85) = 9.508, *p* < 0.0001); training day × group (F(6, 170) = 2.036, ns) (%%%*p <* 0.001, **p < 0.01). Additional significant differences: WT sedentary day 1 versus 2, *p =* 0.04). (C) In open‐field testing *Atxn1* ^154Q/2Q^ mice showed overall lower activity relative to WT mice (*F*(1, 46) = 21.85, *p* < 0.0001) with no effect of exercise (*F*(1, 46) = 0.015, ns). Data represent mean ± SEM. N = 17 (WT sedentary), 13 (WT exercise), 18 (SCA1 sedentary), and 14 (SCA1 exercise). ns = not significant; WT = wild‐type. [Color figure can be viewed at www.annalsofneurology.org]

Motor symptoms were evaluated using an accelerating rotarod, balance beam, and OFT at 16 weeks of age after completing the exercise protocol. On rotarod (Fig 2A), SCA1 sedentary mice performed significantly worse than SCA1 exercise mice on every day of testing (day 1: *p* = 0.03, day 2: *p* = 0.003, day 3: *p* < 0.0001, and day 4: *p* = 0.0002) and worse than WT sedentary mice on days 2 through 4 (day 2: *p* = 0.02, day 3: *p* = 0.005, and day 4: *p* = 0.007).

On the balance beam, SCA1 sedentary mice exhibited longer latency to traverse than SCA1 exercise mice, specifically on days 1 (*p* = 0.009) and 3 (*p* = 0.009) of training (on days 2 and 3, SCA1 exercise mice performed more like both WT cohorts; Fig 2B).

Comparatively, there was no difference in total locomotion between SCA1 sedentary and exercise mice in OFT after 12 weeks of cage wheel running. SCA1 cohorts overall moved less than WT littermates (Fig 2C). There was also no difference in anxiety, measured by percentage of center beam breaks (Supplementary Fig S2).

### Exercise Enables Learning but Does Not Rescue Cognitive Deficits in SCA1 Mice

**FIGURE 3:**
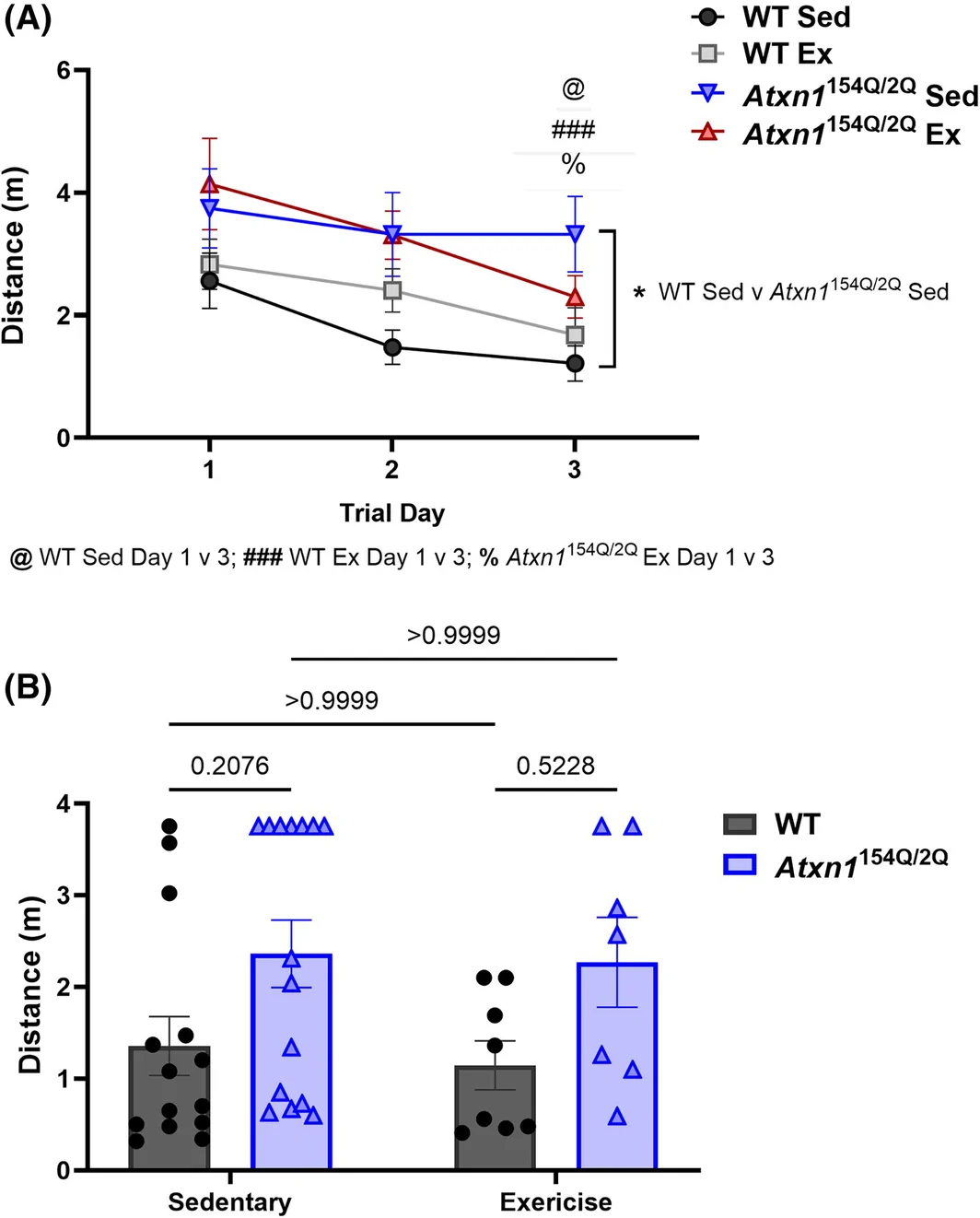
Exercise enables spatial learning in *Atxn1* ^154Q/2Q^ mice without improving memory retention. In the Barnes Maze (A) acquisition phase, WT and exercised *Atxn1* ^154Q/2Q^ mice showed clear learning across training days, demonstrated by reduced path length necessary to find the exit hole, whereas sedentary *Atxn1* ^154Q/2Q^ mice did not improve (training day: (F(1.93, 88.82) = 9.75, *p* = 0.0002); group effect: (*F*(3, 47) = 3.39, *p* = 0.02; training day × group (F(6, 92) = 0.74, ns); @ = 0.049; ### = 0.0005; % = 0.033; * = 0.034. In the (B) probe trial, no exercise‐related differences were observed (*F*(1, 33) = 0.77, ns), yet *Atxn1* ^154Q/2Q^ mice showed overall worse memory retention compared to WT mice (*F*(1, 40), *p* = 0.01) (exercise × genotype (*F*(1, 40) = 0.02, ns). Data represent mean ± SEM. N = 14 (WT sedentary), 12 (WT exercise), 15 (SCA1 sedentary), and 10 (SCA1 exercise; @WT sedentary *p* = 0.04, ###WT exercise *p* = 0.0005). ns = not significant; WT = wild‐type. [Color figure can be viewed at www.annalsofneurology.org]

We next evaluated the impact of exercise on cognitive impairment in this mouse model.^32^ To minimize motor confounds, we measured path distance to the escape tunnel in Barnes Maze testing. By the final day of training (day 3), SCA1 sedentary mice took a significantly longer route than WT sedentary mice (*p = 0.03*) to find the escape tunnel (Fig 3A). Both WT groups and SCA1 exercise mice improved significantly over the training days (day 1 vs 3: WT sedentary *p* = 0.04; WT exercise *p* = 0.0004; SCA1 exercise *p* = 0.04), whereas SCA1 sedentary mice did not. Despite this learning, SCA1 exercise mice did not perform comparably to WT groups by day 3. On the probe test, there was a significant effect by genotype (*p =* 0.01) but not exercise, and post hoc analysis revealed no significant differences between any 2 groups (Fig 3B).

We also analyzed the search strategy (Supplementary Fig S3) to further exclude motor confounds.^27^ Although there was no group effect in search strategy, there was a significant training day effect (*p <* 0.0001). Only SCA1 exercise mice adopted a more efficient strategy (SCA1 day 1 vs day 3: *p* = 0.021).

### Exercise Does Not Impact Cerebellar Degeneration in SCA1 Mice

**FIGURE 4:**
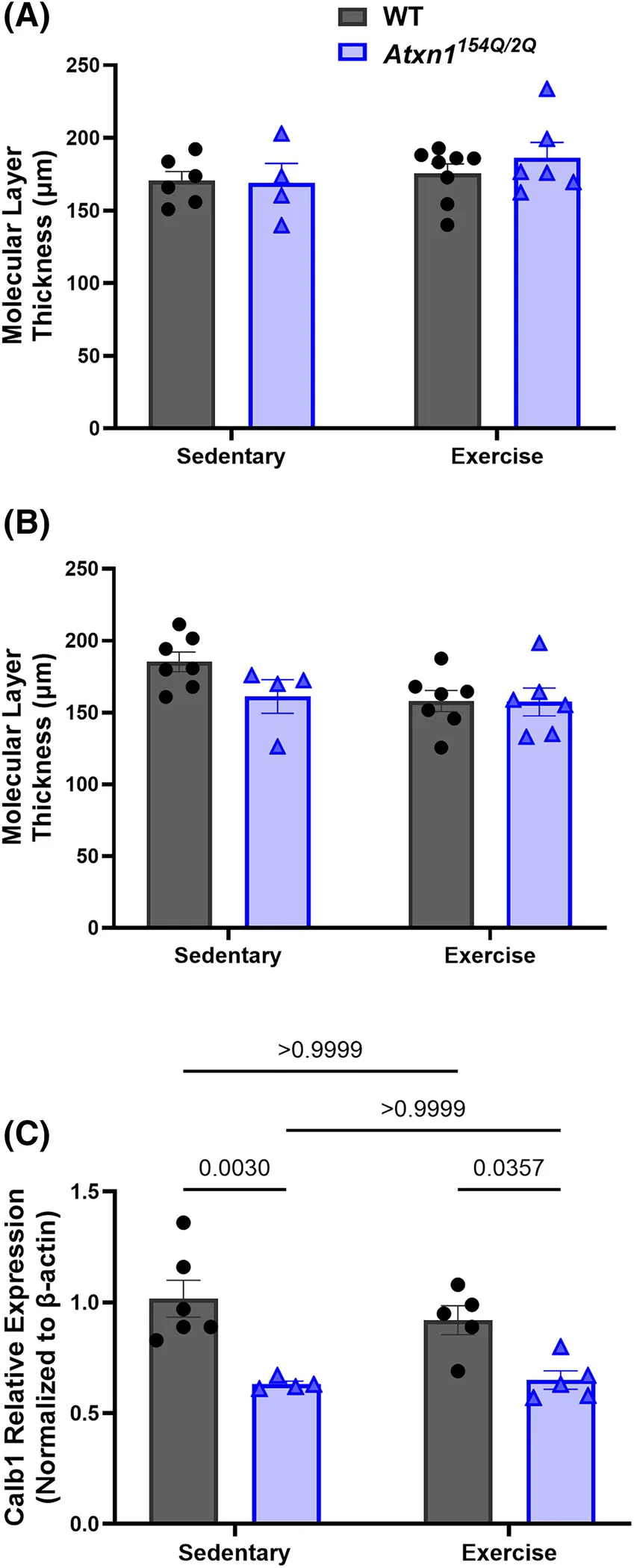
Exercise does not offer structural protection to PC health at 16 to 18 weeks of age. Molecular layer thickness in the (A) anterior and (B) posterior cerebellum did not differ significantly between exercised and sedentary *Atxn1* ^154Q/2Q^ mice (anterior: (*F*(1, 20) = 1.49, ns); posterior: (*F*(1, 20) = 3.13, ns), and no genotype differences (anterior (*F*(1, 20) = 0.27, ns; posterior: (*F*(1, 20) = 2.00, ns), were detected compared to WT mice at this age point. (C) Calbindin gene expression was significantly reduced in *Atxn1* ^154Q/2Q^ mice compared to WT using qRT‐PCR (*F*(1, 16) = 25.45, *p* = 0.0001), although exercise (*F*(1, 16) = 0.37, ns) did not restore expression levels. Data represent mean ± SEM. N = 6 (WT sedentary), 8 (WT exercise), 4 (SCA sedentary), and 6 (SCA exercise). ns = not significant; PC = Purkinje cell; qRT‐PCR = quantitative real‐time polymerase chain reaction; WT = wild‐type. [Color figure can be viewed at www.annalsofneurology.org]

PC degeneration, preceded by dendritic arbor shrinkage, is a hallmark of SCAs including SCA1.^28^ At 16 to 18 weeks of age, MLT was not changed in SCA1 mice, regardless of exercise, in either the anterior or posterior cerebellum (Fig 4A, B, respectively). We did find a significant decrease in calbindin expression by qPCR between WT and SCA1 mice, with post hoc analysis showing similar genotype differences in both sedentary (*p* = 0.003) and exercise (*p* = 0.04) WT versus SCA1 comparisons (Fig 4C). We did not find any difference in calbindin intensity though, in either anterior or posterior hemispheres (Supplementary Fig S4A, B), likely reflecting temporal evolution of structural decay. Consistent with this possibility, we did find a posterior hemisphere loss of molecular layer thickness in 40‐week‐old sedentary SCA1 mice (Supplementary Fig S4C).

### Exercise Minimally Alters Gene Expression Changes in SCA1

**FIGURE 5:**
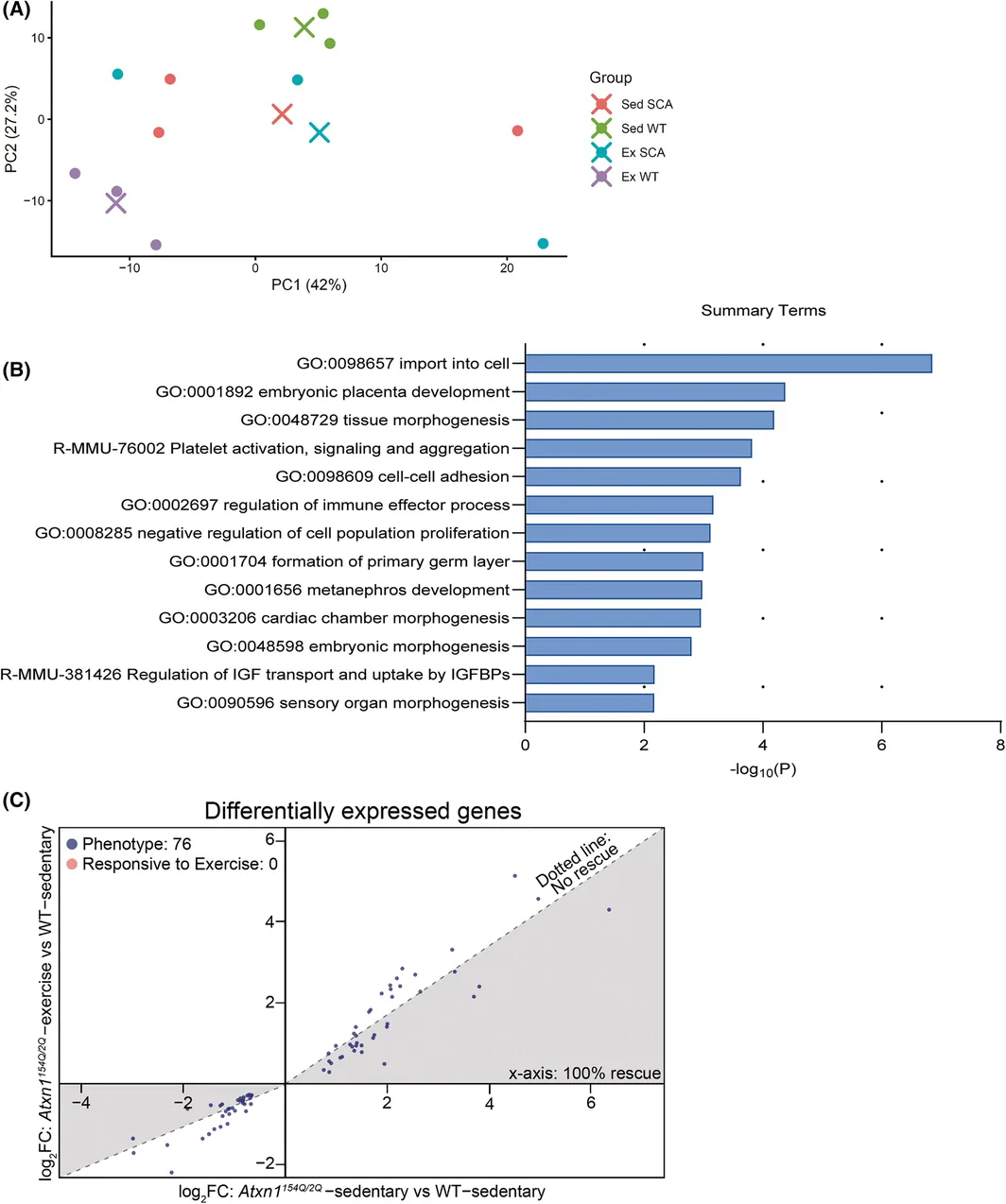
Gene expression profiles in *Atxn1* ^*154Q/2Q*^ mouse cerebellum are not significantly altered by exercise. (A) PCA of differentially expressed genes from *Atxn1* ^154Q/2Q^ sedentary versus WT sedentary (*p*‐adj < 0.05, log2FC > |0.5|), and *Atxn1* ^154Q/2Q^ exercise versus WT sedentary (*p*‐adj < 0.05, log2FC > |0.5|) demonstrates poor clustering and heterogeneity of dispersions across all groups (see Supplementary Table S2). PC axes are scaled in proportion to each principal component's variance explained and X's represent calculated centroids for each experimental cohort. (B) Metascape, GO pathway enrichment analysis of DEGs (*p*‐adj < 0.05, log2FC > |0.5|). (C) DEG rescue analysis revealed minimal changes with exercise in *Atxn1* ^154Q/2Q^ mice. Log_2_FC for WT sedentary versus *Atxn1* ^154Q/2Q^ sedentary is shown on the x‐axis and for WT sedentary versus *Atxn1* ^154Q/2Q^ exercise on the y‐axis. Blue points are phenotypic events log2FC > |0.5|, *p*‐adj < 0.05 – between sedentary WT and *Atxn1* ^154Q/2Q^ mice (n = 79 DEGs); events that cluster around the *x*‐axis show 100% rescue from exercise and events that cluster along the dotted line show no‐rescue from exercise. No genes were responsive to exercise alone [*pink*]). DEGs = differentially expressed genes; FC = fold change; GO = gene ontology; *p*‐adj = adjusted *p* value; PC = Purkinje cell; PCA = principal component analysis; WT = wild‐type. [Color figure can be viewed at www.annalsofneurology.org]

Given the beneficial effects of exercise in SCA1 mice, we used an unbiased approach to identify potential mechanisms contributing to this improvement through bulk RNA sequencing of cerebellar tissue. Initially, we explored changes in DEGs, which recapitulated the reduction in Calbindin (*Calb1*) expression seen by qPCR (see Fig 4C) in sedentary SCA mice compared with sedentary WT mice (log2FC = −0.825, *p*‐adj = 0.0019; Supplementary Table S2). PCA based on genes significantly (*p*‐adj < 0.05, log2FC > |0.5|) impacted by exercise (WT sedentary vs WT exercise, n = 549 DEGs) or by the SCA1 phenotype (WT sedentary vs SCA1 sedentary, n = 76 DEGs) was performed and the observed clustering and spread of samples was assessed with PERMDISP and PERMANOVA. PERMDISP analysis of gene expression data revealed significant differences in group dispersions (*p* = 0.0067), with one sample found to sit closer to another group's centroid than its own, indicating notable overlap between groups. Although PERMANOVA analysis identified significant differences between sedentary WT mice and all other conditions, all other comparisons remained nonsignificant demonstrating a minimal effect of genotype and the interaction of genotype and exercise on differential gene expression. Overall, the PCA plot, PERMDISP, and PERMANOVA analyses revealed wide variance in spread and poor clustering of groups, consistent with a primary dispersion effect and minimal differential gene expression changes between these conditions (Fig 5A, Supplementary Table S4). Pathway enrichment analysis (Metascape Gene Ontology [GO]) of genes differentially expressed between sedentary WT and SCA1 mice revealed limited pathways clearly associated with SCA physiology (Fig 5B and Supplementary Fig S5). When analyzing the effect of exercise on genes differentially expressed between WT sedentary and SCA1 sedentary mice (n = 79 DEGs), it is apparent that there are minimal changes with exercised SCA1 mice, showing similar log2FC values as SCA1 sedentary mice when compared to WT sedentary mice. Some disease relevant genes differentially expressed between sedentary WT and SCA mice, such as *Calb1* and *Trpc3* (Log2FC = −0.669, *p*‐adj = 0.013), which are known to be differentially expressed in the ATXN1[82Q] mouse model,^33^ were not differentially expressed between WT sedentary and SCA exercise mice (*Calb1* Log2FC = −0.403, *p*‐adj = 0.19; and *Trpc3* Log2FC = −0.299, *p*‐adj > 0.99). However, they also showed no significant difference between sedentary and exercised SCA mice (*Calb1* Log2FC = 0.417, *p*‐adj > 0.99; and *Trpc3* Log2FC = 0.365, *p*‐adj > 0.99; see Supplementary Table S2) indicating at most a minor effect of exercise. Other disease relevant genes, such as *Bdnf*, *Egf*, and *Capicua*, showed neither a response to exercise nor a difference between sedentary WT and SCA mice (see Supplementary Table S2). There was also no broader effect of exercise on gene expression changes in SCA1 as no additional genes met significant thresholds between SCA1 exercised and WT sedentary mice to be considered responsive to exercise (Fig 5C). Together, these data demonstrate that exercise has minimal impact on the levels of gene expression in the cerebellum of SCA1 mice.

### Exercise Drives Significant Rescue of Splicing Changes in SCA1

**FIGURE 6:**
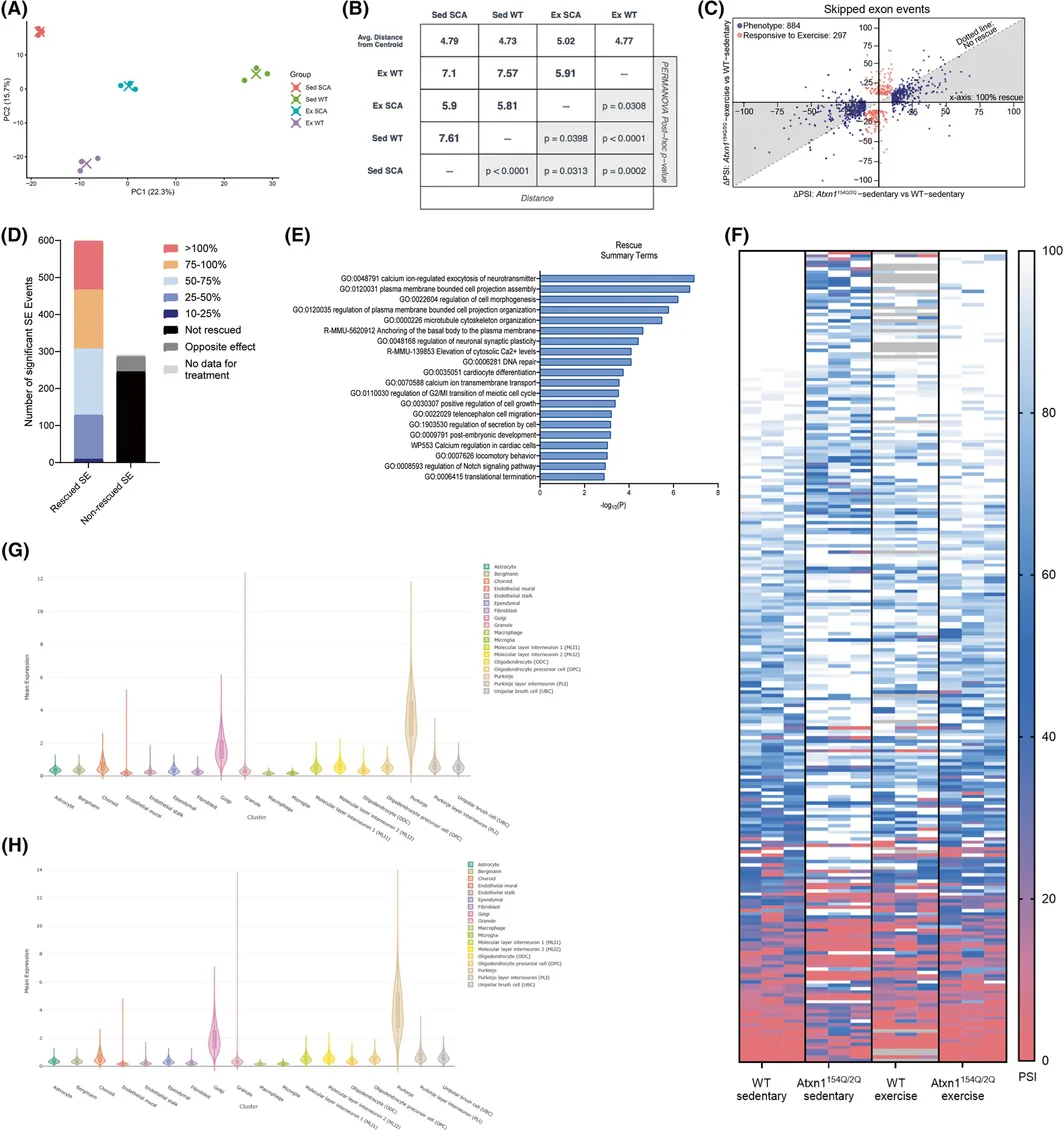
Exercise causes widespread rescue of splicing changes in *Atxn1* ^154Q/2Q^ mouse cerebellum. (A) PCA of mis‐spliced SE events in *Atxn1* ^154Q/2Q^ sedentary versus WT sedentary (FDR < 0.1, ΔPSI > |10%|) and *Atxn1* ^154Q/2Q^ exercise versus WT sedentary (FDR < 0.1, ΔPSI > |10%|) demonstrates robust clustering of all 4 experimental cohorts and clear separation by genotype. PC axes are scaled in proportion to each principal component's variance explained. (B) Distance matrix and PERMANOVA *p* values of alternative splicing data. PSI‐based centroid distances between groups are shown in upper left triangle, with associated post hoc *p* values in lower triangle. The top row of the table indicates the average distance of each sample to its own centroid. Similar values for each group's average distance from its own centroid demonstrates the uniformity of group dispersions. (C) Rescue analysis of 884 significant alternative splicing phenotypic events (*blue*, FDR < 0.1, ΔPSI > |10%| between WT sedentary versus *Atxn1* ^154Q/2Q^ sedentary) with ΔPSI for WT sedentary versus *Atxn1* ^154Q/2Q^ sedentary shown on the x‐axis and ΔPSI for WT sedentary versus exercised *Atxn1* ^154Q/2Q^ shown on the y‐axis. Events that cluster around the *x*‐axis show 100% rescue from exercise and events that cluster along the dotted line show no‐rescue from exercise. There were 297 SE events that were responsive to exercise in *Atxn1* ^154Q/2Q^ mice (*pink*). (D) Bar graph of splicing events (sedentary WT vs *Atxn1* ^154Q/2Q^: FDR < 0.1, ΔPSI > |10%|) broken down by quartiles of percent rescue (*Atxn1* ^154Q/2Q^ sedentary vs *Atxn1* ^154Q/2Q^ exercise: ΔPSI > |10%| and an absolute PSI > |5%|). Nonrescued events are grouped with events of opposite effect and events that were not detected in exercised *Atxn1* ^154Q/2Q^ mice (no data for treatment). (E) Metascape, GO enrichment analysis of genes with rescued SE events (FDR <0.1, ΔPSI > |10%|). (F) Heatmap highlighting 249 SE events dysregulated between WT sedentary and *Atxn1* ^154Q/2Q^ sedentary (FDR <0.1, ΔPSI > |10%|) and significantly rescued by exercise (*Atxn1* ^154Q/2Q^ sedentary versus *Atxn1* ^154Q/2Q^ exercise: FDR < 0.1, ΔPSI > |10%| and an absolute PSI > |5%|), showing splicing rescue in *Atxn1* ^154Q/2Q^ exercise compared with sedentary controls. (G, H) Integration with single‐cell datasets indicated that Purkinje and Golgi cells exhibited the highest expression of genes impacted by SE events (sedentary WT vs *Atxn1* ^154Q/2Q^: FDR < 0.1, ΔPSI > |10%|) (G) and splicing rescue (sedentary WT vs SCA: FDR <0.1, ΔPSI > |10%|; and SCA sedentary vs *Atxn1* ^154Q/2Q^ exercise: ΔPSI > |10%| and an absolute PSI > |5%|) (H). The scaled mean expressions are of 660 genes, WT sedentary versus *Atxn1* ^154Q/2Q^ sedentary (G) and 457 genes with rescued SE events (H), see Methods for details on filtering from individual SE events to individual genes present in the single cell dataset. FDR = false discovery rate; GO = gene ontology; PC = Purkinje cell; PCA = principal component analysis; PSI = change in percent spliced in; SE = skipped exon; SCA = Spinocerebellar ataxia; WT = wild‐type. [Color figure can be viewed at www.annalsofneurology.org]

Aberrant splicing represents an early and prominent disease feature seen in multiple SCA models.^33^, ^34^, ^35^, ^36^ We therefore examined whether exercise impacted splicing. Consistent with previous studies,^29^, ^34^, ^35^ we found that skipped exon events are the predominant class of dysregulated alternative splicing events in SCA1 mice (57.63% of all dysregulated events; FDR < 0.1, ΔPSI > |10%|; Supplementary Fig S6A). We therefore focused our analysis of the transcriptomic effects of exercise on this event class. Interestingly, in WT mice, skipped exon events were also the primary event class regulated by exercise (54.30% of altered events; Supplementary Fig S6B). In contrast to the PCA of DEGs, a PCA based on skipped exon events significantly impacted by exercise (WT sedentary vs WT exercise, n = 823 SEs) or by the SCA1 phenotype (WT sedentary vs SCA1 sedentary, n = 888 SEs) demonstrated clustering and separation between WT and SCA1 exercise and sedentary cohorts (Fig 6A). PERMDISP analysis of PSI values indicated no significant difference in dispersions across groups (*p* = 0.32). PERMANOVA analysis of the same data demonstrates that all genotype and treatment conditions cluster significantly distinctly from each other. Centroid distances indicate that SCA1 exercise mice occupy a broadly intermediate phenotype between all other groups demonstrating a significant impact of both genotype and exercise on alternative splicing with a prominent rescue of splicing in SCA1 exercise mice (Fig 6B).

Further investigation of the effect of exercise on SCA1 mice revealed that of the events dysregulated in SCA1, many showed a smaller difference between SCA1 exercised mice and WT sedentary mice than between sedentary SCA1 and WT mice (Fig 6C). We also detected 297 SE events altered between exercised SCA1 mice and sedentary WT mice that were not dysregulated between sedentary SCA1 and WT mice (see Fig 6B). Interestingly, 32% of these events were either significantly regulated by exercise in sedentary versus exercised WT mice (46 events) or occurred in genes with different exons regulated by exercise (49 events). Of the 888 events dysregulated between sedentary SCA1 and WT mice, 598 (67.34%) were rescued by at least 10%, with 291 events (32.77%) showing >75% rescue toward WT splicing levels; 4 events were not detected in the SCA1 exercised mice and the remaining 290 events were not rescued (Fig 6D). Metascape GO analysis based on the 598 rescued SE events identified pathways heavily associated with SCA physiology, including microtubule cytoskeleton organization and regulation of synaptic plasticity, with calcium ion‐regulated exocytosis of neurotransmitters being the most enriched pathway (Fig 6E). Of the 598 rescued events, 249 were found to be significantly rescued (sedentary vs exercised SCA1 mice FDR < 0.1) representing a strong splicing response to exercise in SCA1 mice (Fig 6F). Integrating our findings with a computational single‐cell atlas of the cerebellum revealed that for genes impacted by mis‐splicing between sedentary WT and SCA and for genes for which exercise rescued mis‐splicing, their average expression was highest in PCs among cell types in the cerebellum (Fig 6G, H).^29^ Together, these data demonstrate a broad transcriptional response to exercise in SCA1 mice driven by robust rescue of dysregulated alternative splicing events in disease relevant pathways.

### *Exercise Rescues Splicing of* Kcnma1

Given both the importance of PC firing to SCA physiology and motor behavior, and our observed rescue of splicing changes in ion channel modules, we explored particularly relevant genes in detail. Aberrant splicing of key ion channel genes in synaptic plasticity (namely *Kcnma1*, *Trpc3*, and *Cacna2d2*) is conserved across multiple SCA murine datasets.^34^ Using an independent cohort of mice from our exercise protocol, we performed PCR across the relevant exons for these genes followed by capillary electrophoresis to quantify the splice isoforms.

**FIGURE 7:**
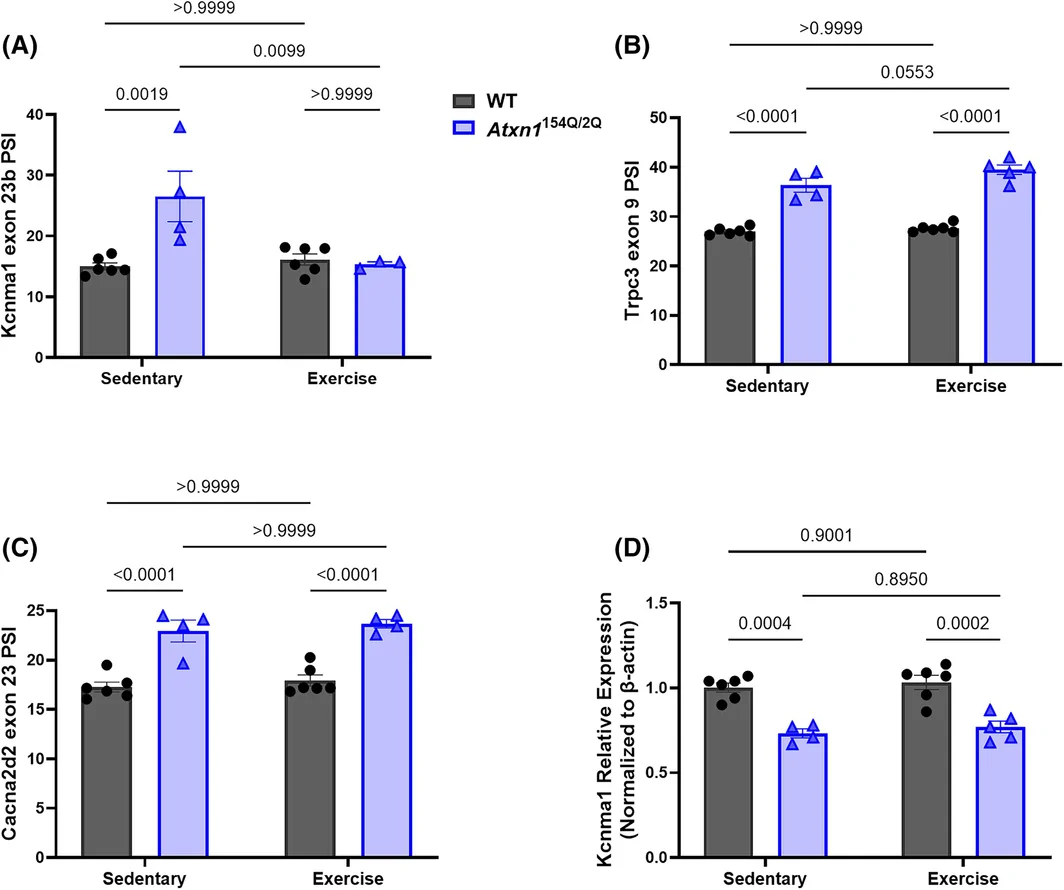
Exercise selectively restores splicing of *Kcnma1*, but not other mis‐spliced circuit‐based genes. (A) *Kcnma*1 showed elevated inclusion of exon 23b in sedentary *Atxn1* ^154Q/2Q^ compared to WT mice (*F*(1, 15) = 7.80, *p* = 0.01), and exercise significantly reduced this inclusion (*F*(1, 15) = 6.78, *p* = 0.02), with a significant genotype × exercise interaction (*F*(1, 15) = 10.16, *p* = 0.006). (B) *Trpc3* (exon 9) showed persistently elevated exon inclusion in both sedentary and exercised *Atxn1* ^154Q/2Q^ mice relative to WT (*F*(1, 17) = 196.1, *p* < 0.0001). A modest exercise effect was also detected (*F*(1, 16) = 6.37, *p =* 0.02) driven by a slightly higher PSI in exercised versus sedentary *Atxn1* ^154Q/2Q^ mice, (genotype × exercise *F*(1, 17) = 2.6, ns). (C) *Cacna2d2* (exon 23) also showed persistently elevated exon inclusion in both sedentary and exercised *Atxn1* ^154Q/2Q^ mice relative to WT (*F*(1, 16) = 73.59, *p* < 0.0001), which was not affected by exercise (*F*(1, 16) = 1.09, ns; genotype × exercise *F*(1, 16) = 0.004, ns). (D) Overall *Kcnma1* expression was significantly decreased in *Atxn1* ^154Q/2Q^ mice compared with WT controls (*F*(1, 17) = 57.04, *p* < 0.0001), with no effect of exercise (*F*(1, 17) = 0.96, ns; genotype × exercise *F*(1, 17) = 0.009, ns). ns = not significant; WT = wild‐type. [Color figure can be viewed at www.annalsofneurology.org]

Confirming prior work, we found significant genotype‐dependent mis‐splicing in *Kcnma1*, *Trpc3*, and *Cacna2d2* (Fig 7A–C).^34^ Exercise did not affect *Trpc3* or *Cacna2d2* splicing. Notably, *Kcnma1* was the only transcript showing a significant rescue in splicing ratios in exercised SCA1 mice, which were fully restored to WT levels. *Kcnma1* encodes for large conductance (BK) voltage‐gated potassium channels, dysfunction of which drives aberrant PC firing in murine models of SCAs.^28^, ^37^, ^38^, ^39^ Importantly, overall *Kcnma1* expression levels themselves did not change with exercise, as SCA1 mice, both exercise and sedentary, expressed significantly lower *Kcnma1* levels compared with WT mice (Fig 7D). Thus, exercise leads to a specific rescue of splicing, but not expression levels, of *Kcnma1*, which underlies SCA pathophysiology.

## Discussion

Cardiovascular exercise is well known to improve clinical and functional outcomes across a range of disorders, including several neurodegenerative diseases.^40^, ^41^ However, the mechanisms driving these neuroprotective effects remain unclear, and even less is known in SCAs specifically. Here, we focused on SCA1 using the well characterized Atxn1^154Q/2Q^ knock‐in mouse model. Previous work reported incomplete motor rescue in this model following a fixed‐speed, 4‐week rotarod exercise protocol.^21^, ^22^ We instead implemented an extended, voluntary 12‐week running paradigm to evaluate both motor and cognitive features of disease progression, complemented by unbiased cerebellar transcriptomics to better characterize the molecular response to aerobic exercise.

After 12 weeks of consistent exercise, SCA1 mice exhibited significant improvements relative to sedentary SCA1 controls, performing more like WT mice on motor coordination tasks. However, this improvement did not extend to overall locomotion, which remained diminished regardless of exercise. These findings suggest that exercise may selectively benefit specific motor domains, particularly balance and coordination, that are clinically relevant to early symptoms and fall risk in patients with SCA1.^42^, ^43^

In line with previous studies, we observed cognitive dysfunction in this SCA1 model.^24^, ^32^, ^44^ Exercised SCA1 mice demonstrated improved learning, although not to WT performance, showing clearer acquisition and more efficient search strategies across training days. However, long‐term reference memory was not restored.^26^ Together, these results imply that exercise in SCA1 may enhance specific cognitive domains, such as decision making, which is linked to prefrontal cortical function, rather than restoring hippocampal‐dependent memory.^45^ Although numerous studies have shown that exercise strongly promotes hippocampal plasticity, early hippocampal atrophy has been reported in this SCA1 model.^44^, ^46^, ^47^, ^48^ Thus, hippocampal damage may not be responsive to the exercise protocol used in this model. It is also worth noting that solo‐housing mice can alter cognitive performance,^49^, ^50^ although we did not find differences in our studies (see Supplementary Fig S1). Nevertheless, our results must be interpreted in that context. Future studies will need to incorporate rigorous testing of multiple cognitive domains and additional brain regions in both solo and group‐housed mice to fully understand the neuroprotective impact of exercise.

Despite finding clear behavioral differences in exercised mice, we did not detect overt neurodegeneration at 16 weeks of age. Although this contradicts previous work, the lack of degeneration was consistent across the anterior and posterior regions, suggesting that early‐stage behavioral deficits reflect impaired physiology rather than measurable structural changes.^25^ Methodological differences in staining or quantification also cannot be ruled out, as genotype‐dependent changes became apparent at a later age (40 weeks; see Supplementary Fig S4C), confirming a progressive disease course.^24^ Additionally, we detected significant reduction of calbindin expression in SCA1 mice compared with WT controls, in agreement with declining PC health preceding structural degeneration.^28^, ^37^, ^51^, ^52^ Notably, aerobic exercise did not alter molecular layer thickness or calbindin expression, indicating that its benefits in SCA1 likely stem from functional or molecular adaptations rather than structural remodeling of PCs at this stage. Murine models of other SCAs have demonstrated anterior predominant atrophy,^53^, ^54^, ^55^ and so it may be that regional PC degeneration is driven by genotype‐specific features, including synaptic aspects due to input from brainstem nuclei. Future work will explore connections between PCs and other cerebellar and brainstem nuclei.

Previous studies showed that exercise benefits in SCA mice may proceed through *Bdnf*, *Egf*, and *Capicua*.^21^, ^22^, ^23^ Building on this work, we used an unbiased transcriptomics approach to explore molecular mechanisms underlying the behavioral rescue observed in our mouse model. We identified minor DEG changes between SCA1 and WT mice, consistent with disease progression at 16 weeks, but these expression differences were not corrected by exercise. Moreover, we did not find significant changes in *Bdnf*, *Egf*, or *Capicua* expression as previously reported,^21^, ^22^, ^23^ suggesting that exercise effects on these pathways are age‐dependent or regulated post‐transcriptionally (see Supplementary Table S2). An age‐dependent effect aligns with findings reporting that only age, not exercise, significantly influences gene expression in cerebellar tissue from WT mice.^56^

In SCA1, early transcriptional pathology is driven by widespread splicing dysregulation prior to differential gene expression.^34^, ^35^ Indeed, aberrant splicing is a hallmark of multiple SCAs (1, 2, 3, and 7), affecting genes involved in microtubule dynamics, transcriptional regulation, and DNA repair.^57^ In our study, the most prominent molecular effect of exercise was rescue of aberrant splicing, with exercised SCA1 and WT mice exhibiting splicing profiles more like each other than their sedentary counterparts. Notably, the cell types with highest expression of genes with prominent splicing changes are the most vulnerable in disease and also showed the highest expression of genes with rescued mis‐splicing events (see Fig 6G, H).

These splicing changes indicate that exercise does not necessarily “normalize” SCA1 splicing toward a WT pattern. Rather, exercise induces broad cerebellar splicing alterations that may reflect a general response to physical activity, not one specific to SCA1. Notably, exercise restored splicing of genes critical for synaptic plasticity and ion homeostasis, especially in Purkinje and Golgi cells. Of note, this broad rescue of mis‐splicing following exercise is consistent with recent intervention human trials in myotonic dystrophy, a CTG repeat expansion disorder in which mis‐splicing drives key disease symptoms, where each individual's mis‐splicing profile from muscle biopsies were rescued following aerobic or strength training.^58^ Other murine studies have also shown splicing changes in skeletal muscle^59^ and brain^60^ with similar training paradigms. Although SCAs are predominantly neurological, skeletal muscle atrophy is noted in SCA1,^61^ and future studies will examine transcriptional patterns in systemic tissues, including skeletal muscle, to ascertain any peripheral contribution to neuroprotective benefits.

Dysregulated ion channel function, most notably of BK channels (encoded by *Kcnma1*), underlies PC firing abnormalities and motor deficits in SCAs^28^, ^37^, ^39^, ^51^, ^62^, ^63^, ^64^ and we observed robust correction of *Kcnma1* missplicing. BK channels regulate afterhyperpolarization and firing regularity,^65^, ^66^ and this splicing correction may underlie the motor improvements in exercised SCA1 mice. Although electrophysiological studies are needed to confirm direct effects on PC firing, our findings highlight splicing—particularly in synaptic and ion‐regulating genes—as a promising therapeutic target.^67^ Aerobic exercise may mitigate disease phenotypes by promoting compensatory splicing patterns rather than reversing all pathogenic events, suggesting that correcting BK channel splicing or activity pharmacologically may be therapeutically beneficial.

This study has several limitations. First, we only assessed mice at one disease stage (16 weeks), leaving open whether exercise benefits persist or are transient. Prior work suggests that at least mortality benefits extend beyond this window.^22^ Future work will further expand upon the importance of exercise timing, including initiation after disease onset (eg, 8–12 weeks in the SCA1 mouse model used here) and cessation of cage‐wheel running prior to symptom onset as in previous studies.^21^, ^22^ Second, the type of exercise may influence the extent of neuroprotection. Whereas aerobic exercise outperforms balance training in humans,^12^ the effects of resistance or even combination training remain unexplored. It may be that a multimodal exercise approach yields the most robust rescue of disease pathology, a testable strategy in our platform. Third, RNA‐seq sample size was limited (n = 3/group), sufficient for robust splicing changes but underpowered for subtle transcriptional or sex‐specific effects. Finally, bulk RNA sequencing captures mixed cell populations and PC‐specific effects may be masked by other cell types. Future studies using single‐cell or cell‐type–specific transcriptomic approaches combined with long‐range sequencing at a high read depth will be necessary to directly characterize the impact of exercise on PCs.

The lack of effective therapies for SCAs demand that we pursue alternative approaches. Although clearly effective, cardiovascular exercise is physically challenging for patients with ataxia. Our findings suggest that exercise‐induced splicing correction—particularly in ion channel modules—may represent a sensitive and targetable mechanism of benefit, independent of overt cerebellar structural changes. This raises the possibility of pharmacomimetic interventions that replicate exercise‐driven molecular effects. Given the conserved benefits of exercise across SCA genotypes, future studies should assess whether splicing correction is a shared adaptive mechanism in other models. Moreover, because exercise engages multiple organ systems, integrating systemic cardiovascular and metabolic profiling will be essential to define dose–response relationships and separate central nervous system (CNS) effects from peripheral contributions.

## Author Contributions

I.S., M.B., D.P., and S.R.S. contributed to the conception and design of the study. I.S., M.B., B.S.N., E.P., B.D.C., M.S., D.P., E.C., T.K.V., H.K.S., and S.R.S. contributed to the acquisition and analysis of data. I.S., M.B., B.S.N., B.D.C., H.L.P., H.K.S., and S.R.S. contributed to drafting the text or preparing the figures.

## Potential Conflicts of Interest

Nothing to report.

## Supporting information


**Supplementary Figure S1.** Solo housing does not affect motor or cognitive performance for WT or SCA1 mice. WT and *Atxn1* ^154Q/2Q^ mice were either solo‐housed or group‐housed from post‐weaning to 16 weeks of age and then phenotyped. (A) There was no effect of genotype *F*(1, 18) = 0.003, ns, housing (*F*(1, 18) = 1.646, ns), or genotype × housing interaction (*F*(1, 18) = 0.15, ns) as determined by 2‐way ANOVA on day 4 of rotarod. (B) In the Barnes maze, there was a significant group effect (*F*(3, 32) = 3.84, *p* = 0.018), specifically seen on day 3, with no effect on training day (*F*(1.91, 60.29) = 2.43, ns) (training day × group (*F*(6, 63) = 0.41, ns). Rotarod: N = 3 (solo housed WT; SCA1), 6 (WT group housed), 10 (SCA1 group‐housed). Barnes Maze: N = 4 (solo/group housed WT; group housed SCA1), and 3 (SCA1 group housed).

**Supplementary Figure S2.** Exercise did not alter anxiety‐like behavior in *Atxn1* ^154Q/2Q^ or WT mice. Anxiety was assessed using the open field test, calculated as the percentage of beam breaks occurring in the center of the chamber relative to total beam breaks. No significant differences were observed between genotypes (*F*(1, 46) = 1.12, ns), exercise conditions (*F*(1, 46) = 1.18, ns), or genotype × exercise interaction (*F*(1, 46) =0.96, ns). Data are mean ± SEM.

**Supplementary Figure S3.** Exercise moderately affects cognitive performance in WT and *Atxn1* ^154Q/2Q^ mice. Exercised *Atxn1* ^154Q/2Q^ mice showed a significant shift from a random to a more direct search strategy (*p* = 0.021), reflected as improved cognitive (BUNS) score in Barnes Maze testing (training day (*F*(1.8,89.79) = 12.49, *p* < 0.0001); group effect (*F*(3, 48) = 1.20, ns); training day × group (*F*(6,96) = 1.086, ns)). Data are mean ± SEM.

**Supplementary Figure S4.** Cerebellar degeneration in *Atxn1* ^154Q/2Q^ mice requires aging. Calbindin intensities by immunofluorescence at 16 weeks show no genotype or exercise difference in either anterior (A) or posterior (B) hemispheres. Anterior: genotype (*F*(1, 16) = 4.00, ns); exercise (*F*(1, 16) = 0.64, ns); genotype × exercise (*F*(1, 16) = 0.90, ns). Posterior: genotype (*F*(1, 16) = 1.25, ns); exercise (*F*(1, 16) = 0.46, ns); genotype × exercise (*F*(1, 16) = 0.85, ns). (C) At 40 weeks, however, there is a reduction in molecular layer thickness in the posterior (*t*(6) = 3.81, *p* = 0.009) cerebellum of *Atxn1* ^154Q/2Q^ mice compared with WT controls, which is not seen in the anterior region (*t* (4) = 1.90, ns). Data are mean ± SEM.

**Supplementary Figure S5.** Top 50 hits through Metascape Gene Ontology Pathway enrichment analysis of DEGs. (A) WT sedentary versus WT Exercise (log2FC > |0.5|, *p*‐adj < 0.05). (B) WT Sedentary versus *Atxn1* ^154Q/2Q^ sedentary mice (log2FC > |0.5|, *p*‐adj < 0.05).

**Supplementary Figure S6.** Partition of splicing events by category. (A) Splicing events dysregulated between WT and SCA1 sedentary mice demonstrates that the largest category are skipped exon (SE) events. (B) In WT mice, exercise predominantly affects SE events. A3SS = Alternative 3′ Splice Site, A5SS = Alternative 5′ Splice Site, MXE = Mutually Exclusive Splicing, RI = retained intron.

**Supplementary Table S1.** RNA integrity (RIN) scores for RNA sequencing samples. * Denotes the 2 samples that required modified library preparation methodology.

**Supplementary Table S2.** Distance matrix and PERMANOVA *p* values of gene expression data. Distances, representing raw counts, between centroids of each group are shown in the upper triangle, with associated post hoc *p* values in the lower triangle shaded in gray. The top row of the table indicates the average distance of each sample to its own centroid. Widespread variation in each group's average distance from its own centroid highlights the heterogeneity of dispersions revealed by the PERMDISP test. Groups in which at least one sample is closer to another group's centroid than its own are highlighted in yellow.

**Supplementary Table S3.** DGE changes for genes of interest in SCA pathophysiology.

**Supplementary Table S4.** Unfiltered DGE changes for all comparisons.

**Supplementary Table S5.** Unfiltered alternative splicing changes for all comparisons.

## Data Availability

All RNA‐seq datasets used in this study will be publicly available through the NCBI Gene Expression Omnibus (GEO) under accession number GSE324036. Any additional data that support the findings of this study are available from the corresponding author upon reasonable request.
