# Supplementary material for: Cardiovascular Exercise Drives Neuroprotection in a Mouse Model of Spinocerebellar Ataxia 1 Via Rescue of Aberrant Splicing
Source: Ann Neurol. Author manuscript; Available in PMC 2026 Aug 19. (PMC13482548; doi:10.1002/ana.78282)
Supplement: Supplementary file 1 [file NIHMS2200424-supplement-Supplemental_Materials.docx]

**Supplemental Materials:**

**SUPPLEMENTAL FIGURE LEGENDS:** 

**Supplemental Figure S1: Solo housing does not affect motor or cognitive performance for WT or SCA1 mice**. WT and *Atxn1*^154Q/2Q ^mice were either solo-housed or group-housed from post-weaning to 16 weeks of age and then phenotyped**(A)** There was no effect of genotype F(1,18) = 0.003, ns),housing (F(1,18) = 1.646, ns), or genotype x housing interaction (F(1,18) = 0.15, ns) as determined by 2-way ANOVA on day 4 of rotarod. **(B)** In the Barnes maze, there was a significant group effect (F(3,32) = 3.84,  *p*= 0.018) , specifically seen on Day 3, with no effect on training day (F(1.91, 60.29) = 2.43, ns) (training day x group (F(6,63) = 0.41, ns)). Rotarod: N = 3 (solo housed WT;SCA1), 6 (WT group housed),10 (SCA1 group-housed). Barnes Maze: N = 4 (solo/group housed WT; group housed SCA1), 3 (SCA1 group housed). 

**Supplemental Figure S2. Exercise did not alter anxiety-like behavior in** ***Atxn1***^**154Q/2Q**^ **or WT mice.** Anxiety was assessed using the open field test, calculated as the percentage of beam breaks occurring in the center of the chamber relative to total beam breaks. No significant differences were observed between genotypes (F(1,46)=1.12, ns), exercise conditions (F(1,46)=1.18, ns), or genotype x exercise interaction (F(1,46)=0.96, ns). Data are mean ± SEM.

 

**Supplemental Figure S3. Exercise moderately affects cognitive performance in WT and** ***Atxn1***^**154Q/2Q**^ **mice.** Exercised *Atxn1*^154Q/2Q ^mice showed a significant shift from a random to a more direct search strategy (*p* = 0.021), reflected as improved cognitive (BUNS) score in Barnes Maze testing (training day (F(1.8,89.79) = 12.49, *p*<0.0001); group effect (F(3,48) = 1.20, ns); training day x group (F(6,96) = 1.086, ns)). Data are mean ± SEM.

 

**Supplemental Figure S4. Cerebellar degeneration in** ***Atxn1***^**154Q/2Q**^ **mice requires aging.** Calbindin intensities by immunofluorescence at 16 weeks show no genotype or exercise difference in either anterior (**A**) or posterior (**B**) hemispheres. Anterior: genotype (F(1,16) = 4.00, ns); exercise (F(1,16) = 0.64, ns); genotype x exercise (F(1,16) = 0.90, ns). Posterior: genotype (F(1,16) = 1.25, ns); exercise (F(1,16) = 0.46, ns); genotype x exercise (F(1,16) = 0.85, ns). (**C**) At 40 weeks however, there is a reduction in molecular layer thickness in the posterior (t(6) = 3.81, *p* = 0.009) cerebellum of *Atxn1*^154Q/2Q^ mice compared to WT controls, which is not seen in the anterior region (t(4) = 1.90, ns).  Data are mean ± SEM.

 

**Supplemental Figure S5: Top 50 hits through Metascape Gene Ontology Pathway enrichment analysis of DEGs. (A**) WT sedentary versus WT Exercise (log2FC >|0.5|, padj < 0.05). (**B**) WT Sedentary versus *Atxn1*^154Q/2Q ^sedentary mice. (log2FC >|0.5|, padj < 0.05).  

**Supplemental Figure S6: Partition of splicing events by category.** (**A**) Splicing events dysregulated between WT and SCA1 sedentary mice demonstrates that the largest category are skipped exon (SE) events. (**B**) In WT mice, exercise predominantly affects SE events. A3SS – Alternative 3’ Splice Site, A5SS – Alternative 5’ Splice Site, MXE – Mutually Exclusive Splicing, RI – retained intron.

**Supplemental Table S1: RNA integrity (RIN) scores for RNA sequencing samples. *** Denotes the two samples that required modified library preparation methodology. 

**Supplemental Table S2**: **Distance matrix and PERMANOVA p-values of gene expression data.** Distances, representing raw counts, between centroids of each group are shown in the upper triangle, with associated post-hoc *p*-values in the lower triangle shaded in gray. The top row of the table indicates the average distance of each sample to its own centroid. Widespread variation in each group’s average distance from its own centroid highlights the heterogeneity of dispersions revealed by the PERMDISP test. Groups in which at least one sample is closer to another group’s centroid than its own are highlighted in yellow.

**Supplemental Table S3: DGE changes for genes of interest in SCA pathophysiology.**

**Supplemental Table S4: Unfiltered DGE changes for all comparisons** 

**Supplemental Table S5: Unfiltered Alternative Splicing Changes for all comparisons**
